# Incidence of marginal ulcer after one anastomosis gastric bypass versus Roux-en-Y gastric bypass: a comparative study

**DOI:** 10.1186/s12893-026-03559-y

**Published:** 2026-02-20

**Authors:** Ahmed Abdelsalam, Ahmed Fahmy, Ahmed Saqr, Sherkawi Elsayed, Ahmed Mohamed Salah Eldden Othman Elansary

**Affiliations:** General Surgery Department, Kasr Alainy Medical School, Cairo University, Cairo, Egypt

**Keywords:** Roux-en-Y gastric bypass, One-anastomosis gastric bypass, Marginal ulcer

## Abstract

**Background:**

Although Roux-en-Y gastric bypass (RYGB) has proven to be a safe and effective choice for long-standing weight loss, associated complications have been described. Among them, marginal ulcer (MU) has been recognized as one of the more significant postoperative complications. One-anastomosis gastric bypass (OAGB) has currently been the third most common procedure overall in the world. Similar to RYGB, concerns have emerged for MU risk after OAGB. This study aimed to compare the incidence of MU after RYGB versus OAGB.

**Methods:**

This is a cross-sectional study that included 62 adult patients who underwent RYGB or OAGB and were followed up for a period of two years. The included patients underwent a complete general examination and upper gastrointestinal endoscopy.

**Results:**

The prevalence of MU in RYGB patients was 19.4%, whereas in OAGB patients, it was markedly lower at 3.2%, with a statistically significant difference (*p* = 0.045). In the multivariate analysis, the presence of diabetes and the type of surgery (RYGB) were significant predictors for MU.

**Conclusion:**

Marginal ulcers occurred at a relatively high rate after bypass surgery. OAGB demonstrated a significantly lower incidence of MUs compared to RYGB. Type 2 diabetes mellitus and the RYGB procedure were significant predictors of MU after adjusting for the confounding factors.

**Supplementary Information:**

The online version contains supplementary material available at 10.1186/s12893-026-03559-y.

## Introduction

Obesity has been an epidemic impacting human health all over the world. It has been estimated that, by 2025, more than one billion individuals will have obesity, and 177 million will have severe obesity if the present trends of obesity continue [1].

For the long-term treatment of severe obesity, bariatric surgery remains the most established and evidence-supported treatment. While lifestyle modifications and pharmacotherapy can provide benefits, their long-term effectiveness is often limited [2]. Among bariatric procedures, the Roux-en-Y gastric bypass (RYGB) is the second most frequently performed bariatric procedure, after sleeve gastrectomy [3]. Although Roux-en-Y gastric bypass (RYGB) has proven to be a safe and effective option for achieving long-term weight loss, associated complications have been reported [4]. Among them, marginal ulcer (MU) has been recognized as one of the more significant postoperative complications [5].

A marginal ulcer has been reported to occur at an incidence ranging from 0.6% to 25%. Its pathophysiology is not fully understood, but variable risk factors, such as acid exposure, ischemic changes, smoking, and infection with Helicobacter pylori, have been implicated as contributing factors to MU [6].

The clinical presentation widely varies. It could be clinically silent or associated with vague abdominal symptoms. Complications such as perforation and bleeding are not uncommon, with described percentages of 8.2% and 9.5%, respectively [6, 7].

One-anastomosis gastric bypass (OABG) has currently been the third most common procedure overall in the world, with an exponential increase in the rate of performing OAGB during the previous 5 years [8]. Similar to RYGB, concerns have emerged for MU risk after OAGB [9].

To date, there is scarce evidence available regarding the incidence of MU after OAGB versus RYGB. This study aimed to assess the incidence of MU following both surgical procedures and to determine the potential risk factors for the occurrence of MU.

A significant limitation of earlier comparative studies is that endoscopic assessment was usually performed only when patients presented with symptoms, potentially leading to a gross underestimation of the true incidence of marginal ulcers. The current investigation addressed that limitation by performing routine surveillance endoscopy on all patients, regardless of symptoms. This enabled the identification of both symptomatic and asymptomatic MUs, yielding a more appropriate comparison between RYGB and OAGB regarding ulcer incidence and insights that symptom-driven endoscopic assessments do not readily capture.

## Patients and methods

This is a cross-sectional retrospective study with prospective follow-up that included patients with obesity who underwent gastric bypass surgery as a bariatric procedure at our institution.

• Ethics Approval: The study was conducted after receiving approval from the institutional ethics committee and was in accordance with the Declaration of Helsinki.

• Informed Consent: Informed written consent was obtained from all patients before they were enrolled in the study.

The sample size of the current study was calculated using a power of 80% and 0.05 alpha error and was based on the described incidence of MU in patients who underwent RYGB and OAGB (2.7% vs. 5%, respectively) [10]. A sample of 62 patients (31 in each group) was required.

Patients included in this study were identified from a prospectively maintained database of consecutive individuals who underwent RYGB or OAGB as a primary bariatric procedure at least two years prior, performed by surgeons of comparable experience using standardized techniques. We excluded patients with severe debilitating diseases, autoimmune conditions, or those on continuous corticosteroid therapy. Additionally, patients with incomplete follow-up data extending to at least two years post-surgery were excluded.

All eligible patients were contacted and invited to participate in a follow-up screening with esophagogastroduodenoscopy (EGD), regardless of whether they were experiencing gastrointestinal symptoms.

All endoscopies were performed by an independent endoscopist who was not involved in the surgical care of the patients and was blinded to the study hypothesis. Those who responded to this recall and consented to undergo EGD were included in the study. Recruitment continued until the calculated sample size for each group was achieved. During the study period, 322 bypass procedures were performed at our institution. Among 73 eligible patients contacted, 62 responded to the phone call, and all agreed to participate, resulting in their enrollment in the study.

We obtained informed written consent from all patients included in the study before enrolling them.

The medical files of the study patients were screened for preoperative, operative, and follow-up data, including full history regarding the history of smoking, drug intake, chronic diseases, medications, operative events, and postoperative complications. In addition, general clinical examination data, including anthropometric measurements with assessment of body mass index (BMI), percentage of excess weight loss (EWL%), and percentage of total weight loss (TWL%), were collected [11]. Remission of comorbidities was judged per the criteria proposed by the American Society for Metabolic and Bariatric Surgery [12].

All patients underwent an EGD examination that was performed by an independent endoscopist under conscious sedation. After routine preparation, the flexible endoscope (Olympus, Centre Valley, PA, USA) was inserted through the mouth and advanced through the esophagus, pouch, and the afferent and efferent loops (in RYGB), or the alimentary limb (in OAGB). A systematic examination was performed to assess the gastric pouch, anastomotic sites, and staple lines for signs of marginal ulcers, including erythema, erosion, or ulceration. The endoscopist measured all gastric pouch dimensions using an articulating measuring instrument, introduced through the working channel of a flexible endoscope. Targeted biopsies were performed for any suspicious lesions, ulcerations, or erythematous areas to confirm the diagnosis and rule out other pathologies. The findings were documented comprehensively, including the location, size, and characteristics of any identified marginal ulcers. High-quality images and video recordings were captured for accurate documentation.

### Outcome measures

The primary outcome of this study was the incidence of MU after OAGB compared to RYGB. The secondary outcomes included the characteristics and clinical presentation of patients who developed MU, as well as the risk factors for MU occurrence.

### Statistical analysis

The study’s data were analyzed using version 28.0 of the SPSS software (Armonk, NY: IBM Corp). Data were expressed as numbers and percentages or as mean ± standard deviation/median (interquartile range) and compared using the chi-square test/z-test for proportions or an independent t-test/Mann-Whitney test accordingly. Binary logistic regression analysis was performed to identify predictors of MU occurrence. Clinically relevant variables with a p-value of less than 0.1 in the univariate analysis were incorporated in a multivariate regression analysis. The significance of the obtained results was judged at the 5% level.

## Results

This study included 62 adult patients who underwent primary RYGB (*n* = 31) or primary OAGB (*n* = 31) between January 2020 and December 2021 and were assessed for the occurrence of MU two years after surgery.

The baseline characteristics of the study patients are delineated in Table 1. The mean ages of patients were 37.2 ± 8.2 years and 36 ± 5.5 years in the RYGB and OAGB groups, with no statistically significant difference (*p* = 0.505). The gender distribution revealed that the majority of patients in both the RYGB and OAGB groups were female, comprising 83.9% and 80.6% of the respective cohorts, with no statistically significant differences (*p* = 0.740). The mean baseline BMI in the RYGB patients was 52.4 ± 11 kg/m2, and the respective mean value in the OAGB was 53.2 ± 10.5 kg/m2, with a statistically non-significant difference (*p* = 0.779).

**Table 1:** Sociodemographic and clinical data of the study patients

Variable	RYGB (*n* = 31)	OAGB (*n* = 31)	*p*-value
Age (year)	37.16 ± 8.24	35.97 ± 5.5	0.505^a^
Sex			
Female	26 (83.9%)	25 (80.6%)	0.740^b^
Male	5 (16.1%)	6 (19.4%)	
Comorbidities			
T2DM	8 (25.8%)	6 (19.4%)	0.544^c^
Hypertension	11 (35.5%)	9 (29.0%)	0.587^c^
Dyslipidemia	22 (71.0%)	20 (64.5%)	0.587^c^
Baseline BMI (Kg/m2)	52.44 ± 11.02	53.21 ± 10.46	0.779^a^
Surgery Time (mins)	114.2 ± 21.6	102.6 ± 14.7	0.165^a^
Pouch Length (cm)	8.1 ± 2.6	17.35 ± 1.76	< 0.001^a*^
Early Postoperative Complications	2 (6.5%)	1 (3.2%)	0.554^b^
Re-intervention	1 (3.2%)	1 (3.2%)	1.0^b^
Postoperative BMI (kg/m2)			
6-month	40.3 ± 7.2	38.3 ± 6.6	0.259^a^
1-year	36.5 ± 8.4	33.2 ± 7.1	0.1^a^
2-year	31.2 ± 7.6	27.8 ± 6.9	0.07^a^
EWL%			
6-month	47.6 ± 10.4	51.7 ± 11.6	0.148^a^
1-year	61.9 ± 17.5	67.8 ± 12.8	0.135^a^
2-year	73.5 ± 17.9	79.7 ± 20.1	0.205^a^
TWL%			
6-month	19.4 ± 7.6	21.9 ± 6.5	0.169^a^
1-year	24.1 ± 7.7	27.1 ± 7.3	0.121^a^
2-year	31.7 ± 6.6	33.5 ± 6.3	0.276^a^
2-year Comorbidities Remission Rate			
T2DM	5/8 (62.5%)	4/6 (66.7%)	0.872^b^
Hypertension	9/11 (81.8%)	8/9 (88.9%)	0.660^b^
Dyslipidemia	20/22 (90.9%)	19/20 (95%)	0.607^b^
Incidence of MU	6 (19.4%)	1 (3.2%)	0.045^b*^
Number of MU			
Single	4 (66.7%)	0 (0%)	0.212^b^
Multiple	2 (33.3%)	1 (100%)	

*T2DM* Type 2 diabetes mellitus, *BMI* Body mass index, *EWL* Excess weight loss, *TWL* Total weight loss, *MU* Marginal ulcer, *PPI* Proton pump inhibitors, *NSAIDs* Non-steroidal anti-inflammatory drugs

^a^ t, t value of the student-t test, ^b^ χ2, Chi-square test, ^c^ Z, Z test for proportion

*Statistically significant

Both groups showed comparable prevalences of associated medical complications. Type II Diabetes Mellitus (T2DM) was prevalent in 25.8% of the RYGB group and 19.4% of the OAGB group (*p* = 0.544). Hypertension was predominant in 35.5% and 29.0% of patients in the RYGB and OAGB groups, respectively (*p* = 0.587), and dyslipidemia was prevalent in 71.0% and 64.5% of the two groups, respectively (*p* = 0.587) (Table 1).

The median surgery time (min) for RYGB was higher than that of OAGB 102 vs. 92), without statistical significance (*p* = 0.554). In terms of pouch length (cm), RYGB had a median pouch length of 7.1, while OAGB presented a significantly larger median pouch length of 17 (*p* < 0.001).

The early postoperative complications rate in the RYGB group was 6.5% (two patients; one case of leakage that required re-intervention and stent placement, and one case of intra-abdominal bleeding that was managed conservatively), while that of the OAGB group was 3.2% (one case of intra-abdominal bleeding necessitating exploration and drainage). The p-value of 0.554 indicates that there is no statistically significant difference.

Non-statistically significant higher weight loss values were seen in the OAGB group during all follow-up time points (*p* > 0.05). Regarding the 2-year comorbidities remission rates, RYGB and OAGB demonstrated T2DM remission rates of 62.5% and 66.7%, respectively (*p* = 0.872). For hypertension remission, RYGB and OAGB exhibited rates of 81.8% and 88.9%, respectively (*p* = 0.660). The remission rates for dyslipidemia were 90.9% and 95%, respectively, in the two groups (*p* = 0.607) (Table 1).

The prevalence of MU in RYGB patients was 19.4%, whereas in OAGB patients, it was significantly lower (3.2%), with a statistically significant difference (*p* = 0.045) (Table 1). Comparing patients with MUs to those who did not develop MUs revealed a statistically significant difference in the incidence of abdominal symptoms, including epigastric pain, with 57.1% of patients in the MU group experiencing this symptom compared to 12.7% in the non-MU group. Other symptoms, such as vomiting, hematemesis, and melena, were exclusively observed in the MU group but not in the non-MU group. A substantial proportion of patients without MUs (87.3%) reported no abdominal symptoms, compared to 28.6% in the MU group, indicating a significant difference in symptomatic presentation (*p* < 0.001) (Table 2).

**Table 2:** The incidence of postoperative marginal ulcer, associated symptoms and potential risk factors

	MU (*n* = 6)		Non-MU (*n* = 25)		*p*-value
	Count	%	Count	%	
Abdominal symptoms					
Epigastric pain	3	50.0%	4	16.0%	0.002*
Vomiting	1	16.7%	0	0%	
Hematemesis	1	16.7%	0	0%	
Melena	1	16.7%	0	0%	
None	2	33.3%	21	84.0%	
PPI use	4	66.7%	4	16.0%	0.011*
Potential risk factors					
Smoking	6	100.0%	10	40.0%	0.008^*^
Cannabis use	2	33.3%	3	12.0%	0.202^c^
Chronic NSAIDs use	1	16.7%	0	0%	0.038*^c^
H-pylori infection	0	0%	3	12.0%	0.372^c^
	Mean ± SD		Mean ± SD		
Pouch length (cm)	12.7 ± 2.07		7.0 ± 1.15		< 0.001^a*^

*MU* Marginal ulcer, *PPI* Proton pump inhibitors, *NSAIDs* Non-steroidal anti-inflammatory drugs

^b^ χ^2^, Chi-square test, ^c^ Z, Z test for proportion

*Statistically significant

Proton pump inhibitor (PPI) use was significantly higher in the MU group (71.4%) compared to the non-MU group (23.6%), with a p-value of 0.009. All patients with MU were smokers (100.0%) compared to 32.7% in the non-MU group (*p* < 0.001). Cannabis use appears more common in the MU group (28.6%) than in the non-MU group (7.3%), but the difference is not statistically significant (*p* = 0.072). Chronic use of NSAIDs is significantly higher in the MU group (14.3%) compared to none in the non-MU group, with a p-value of 0.005. Infection with Helicobacter pylori did not appear to be significantly associated with marginal ulcers in this cohort (*p* = 0.317). The median pouch length was significantly longer in the MU group (12.9 cm) compared to the non-MU group (6.4 cm), with a highly significant difference (*p* < 0.001) (Table 2).

The univariable binary logistic regression analysis for potential predictors of MU occurrence in patients who underwent bypass surgery revealed that the presence of T2DM, epigastric pain, and PPI use had statistically significant associations with MU occurrence (*p* = 0.002, 0.01, and 0.02, respectively). There was a marginal trend toward the association of RYGB with the occurrence of MU (*p* = 0.076 (Table 3). In the multivariate analysis, the presence of T2DM and the type of surgery stood out as significant predictors (*p* = 0.013 and 0.045, respectively) (Table 4).

**Table 3:** Univariable binary logistic regression analysis for the predictors of MU occurrence

Variable	B	SE	*p*-value	OR	95% CI
Age	-0.043	0.058	0.452	0.957	[0.855, 1.072]
Sex	1.483	0.824	0.123	4.406	[1.68, 23.504]
T2DM	3.562	1.146	0.002*	35.25	[3.731, 333.041]
Hypertension	0.517	0.818	0.527	1.676	[0.338, 8.324]
Dyslipidemia	-0.517	0.818	0.527	0.596	[0.12, 2.962]
Baseline weight	-0.006	0.021	0.777	0.994	[0.953, 1.036]
Baseline Height	0.024	0.041	0.558	1.025	[0.945, 1.111]
Baseline BMI	-0.065	0.045	0.152	0.938	[0.858, 1.024]
Surgery type	1.974	1.114	0.076	7.200	[0.812, 63.854]
Surgery Time	-0.01	0.019	0.604	0.990	[0.954, 1.028]
Pouch size	-0.012	0.079	0.881	0.988	[0.847, 1.153]
Epigastric pain	2.213	0.079	0.01*	9.143	[1.68, 49.75]
Vomiting	23.418	40192.962	1.000	1.48 × 10^10^	[0.0, -]
GIT bleeding	23.824	23205.420	0.999	2.22 × 10^10^	[0.0, -]
PPI use	2.089	0.895	0.020*	8.077	[1.398, 46.659]
Smoking	20.584	6201.910	0.997	0.86 × 10^10^	[0.0, -]
Cannabis use	1.629	0.985	0.098	5.100	[0.74, 35.135]
Chronic NSAIDs use	23.418	40192.991	1.000	1.48 × 10^10^	[0.0, -]
H-pylori infection	-19.278	15191.520	0.999	0.000	[0.0, -]

*T2DM* Type II Diabetes Mellitus, *BMI* Body Mass Index, *GIT* Gastro Intestinal Tract, *PPI* Proton Pump Inhibitor, *NSAIDS* Non-Steroidal Anti Inflammatory Drugs

**Table 4:** Multivariate binary logistic regression analysis model for the predictors of MU occurrence

Variable	B	SE	*p*-value	OR	95% CI
T2DM	3.23	1.296	0.013*	25.115	[1.997, 321.355]
Surgery type	2.774	1.114	0.045*	16.021	[1.061, 241.986]
Epigastric pain	0.408	1.664	0.806	1.504	[0.058, 39.192]
PPI use	1.291	1.259	0.305	3.637	[0.308, 42.926]
Cannabis use	-0.44	2.075	0.832	0.641	[0.011, 37.597]

*MU* Marginal Ulcer, *T2DM* Type II Diabetes Mellitus, *PPI* Proton Pump Inhibitors

## Discussion

While obesity can be managed conservatively with medical treatment and a change of lifestyle, surgical solutions are the most established and evidence-supported treatment [13]. In 2018, Roux-en-Y gastric bypass procedures represented 29.3% of the total bariatric procedures performed, according to the latest report from the International Federation for the Surgery of Obesity and Metabolic Disorders. One-anastomosis gastric bypass comes just after RYGB, being now the third most common procedure worldwide [14].

Marginal ulcers (MUs), which typically occur at or near the gastrojejunal anastomosis, are recognized complications of gastric bypass and impose the risk of significant morbidity [5]. Few head-to-head comparisons currently exist assessing the difference between RYGB and OAGB in the incidence of MU. Of these, to our knowledge, this is the first study to compare the incidence of MU after both surgeries based on endoscopic screening.

Notably, the overall rate of MU in the current study was 11.3% (19.4% in the RYGB group and 3.2% in the OAGB group). This high rate could also be attributed to the small cohort and the routine surveillance using upper Gastro-Intestinal Tract GIT endoscopy of all patients, regardless of whether they were symptomatic or not, beyond the 2-year follow-up. Our high rates were consistent with the study by Baksi et al. (2020), who found a MU rate of 9.5% at the 1-year follow-up screening using an upper gastrointestinal GI endoscope [9]. Incidence rates reported in other studies by Musella et al. [15] and Kular et al. [16] were lower than those in the present study, ranging from 0.2% to 4%. These studies utilized endoscopy use for only symptomatic patients. This could underestimate the actual incidence of MU.

Our study revealed that RYGB was associated with a significantly higher prevalence of MU, a finding further supported by the regression analysis. This aligns with reports by Scott-Conner [17], who declared that OAGB was expected to be less frequently associated with MU than RYGB due to the mixing of gastric juice, which is the primary culprit in the MU formation, with bile, duodenal Brunner’s gland secretions, and alkaline pancreatic secretions. The Roux reconstruction was assumed to be more ulcerogenic owing to the total diversion of the alkaline secretions away from the gastrojejunal anastomosis. In the recent review of Salame et al. [5], it was described that, after RYGB, the ingested food bypasses the antrum, and therefore the stimulation of the antrum and the release of gastrin are reduced. Nonetheless, the mucosal lining of the stomach remains capable of responding to hormonal and vagal stimulation, thereby maintaining the acidic environment. Lacking the jejunal mucosa to the buffering mechanisms makes it vulnerable to the effects of the gastric secretions. It has been believed that the pepsin local activation in the mucosa of the jejunum is elicited by the gastric secretions’ acidity. These alterations trigger the MU formation. In addition, the occurrence of gastro-gastric fistula following RYGB has been associated with a reduction in gastric pouch pH, as the acids secreted from the remaining gastric tissue pass through the fistula and into the pouch, contributing to the occurrence of MU.

There was a substantial increase in the likelihood of MUs in patients with T2DM. Its effect was confirmed by being still statistically significant in the multivariate analysis. Our results were in line with those of previous studies, which have described a significant association between T2DM and MU [6, 18–22]. Several mechanisms were proposed to link T2DM to the MU risk, such as the Diabetes Mellitus DM-induced microangiopathy and delayed healing of wounds [18]. Therefore, it is crucial to ensure control of DM to combat MU development after surgery.

In this study, the ongoing use of PPIs exhibited a significant association with the presence of MU. This could be explained by the associated reflux symptoms that cause epigastric pain and prompt patients to use PPIs. This finding was observed by Bekhali and Sundbom, who showed that enduring use of PPI was significantly related to the occurrence of MU [23].

Additionally, this study exhibited that smoking exhibited a strong and significant association with marginal ulcers. This finding is consistent with a molecular study by Li et al., which reported that smoking can result in a reduction in the blood supply to the gastrointestinal mucosa, inhibition of cell renewal, interference with the mucosal immune system, and death of the mucosal cells [24]. Experts recommend smoking cessation for a minimum of 6 weeks before bariatric surgery. Refrain from smoking for at least six weeks preoperatively [25].

The association between PPI use and MU observed in univariate analysis likely reflects reverse causality and confounding by indication. Patients who developed early epigastric symptoms or reflux-often early manifestations of MU-were more likely to receive empiric PPI therapy prior to endoscopic confirmation. Thus, PPI use reflects symptom-driven prescribing rather than a causal risk factor in ulcer formation. Symptomatic overlap, along with interactions among smoking, NSAID use, and pouch characteristics, likely accounts for why PPI use lost statistical significance in the multivariate model.

Chronic NSAID use was another significant risk factor for MU in the current study, with 16.7% of patients in the MU group using NSAIDs chronically compared to none in the non-MU group. This association can be attributed to several factors: NSAIDs inhibit cyclooxygenase, leading to reduced prostaglandin synthesis, which is crucial for maintaining the protective mucosal barrier in the gastrointestinal tract. Additionally, NSAIDs cause direct mucosal damage and impair the healing of existing lesions, making the gastric lining more susceptible to injury. The compounded effects of altered gastric physiology post-RYGB, such as changes in acid secretion and increased bile exposure, may exacerbate the risk. Patients using NSAIDs often have chronic conditions that necessitate their use, further increasing their vulnerability to ulcers. These findings emphasize the need for alternative pain management strategies and the potential use of gastroprotective agents in RYGB patients to mitigate the risk of MU.

Our findings are in line with the study of Portela et al. [26], who described NSAID use as a well-established risk factor for marginal ulceration after RYGB. The same finding was reported by Di Palme et al. [22].

Whereas smoking and chronic NSAID use were strongly associated with MU in univariate analysis, neither behavioral factor remained significant in the multivariate model. Collinearity with surgery type and pouch length, which both strongly influenced MU risk and shared explanatory variance with these behavioral factors, likely contributed to this effect. Furthermore, the statistical power to detect multiple independent predictors was dampened by the limited number of MU events. Notably, the direction of effect for both smoking and NSAID use remained positive, indicating clinical relevance is sustained despite a lack of attainment of independent significance following adjustment.

Anatomical factors also play a crucial role, with the mean pouch length being significantly longer in the MU group compared to the non-MU group. This suggests that a longer pouch may be a substantial risk factor for the development of marginal ulcers. Several reasons can explain this association: A longer pouch may result in increased exposure of the gastric mucosa to gastric acids and bile, leading to greater mucosal irritation and damage. Additionally, a longer pouch might slow gastric emptying, causing food and acidic contents to remain in contact with the mucosal lining for extended periods, thereby increasing the risk of ulceration. The increased surface area in a longer pouch also means there is more mucosa that can be affected by the stress and strain of peristalsis and other mechanical forces.

This finding is consistent with previous studies. For instance, Salame et al. [5] identified larger pouch sizes as a significant risk factor for MU, likely due to increased exposure to gastric acids and bile and potential delayed gastric emptying. Additionally, a recent study by Baldwin et al. [27] highlighted that larger pouches can impair mucosal healing due to compromised blood supply.

Our findings are consistent with the recent systematic review and meta-analysis by Beran et al., which represents one of the most comprehensive evaluations of marginal ulcer risk after gastric bypass to date. Their analysis identified several key predictors of MU—most notably smoking, NSAID use, diabetes mellitus, and pouch-related anatomical factors—many of which were corroborated in our cohort. Importantly, similar to our results, Beran et al. highlighted the substantial heterogeneity across studies arising from variations in surgical technique, limb lengths, postoperative surveillance practices, and definitions of MU. This reinforces the relevance of our study’s protocol, which utilized routine endoscopic screening to ensure uniform MU detection and minimize symptom-driven bias. The alignment between our findings and this recent high-quality synthesis underscores the validity of the identified risk factors while also emphasizing the need for standardized diagnostic and follow-up protocols across bariatric centers [28]. 

Regarding weight loss, a comparison of the percentage of excess weight loss (EWL) and total weight loss (TWL) between the two surgeries at different time points postoperatively showed the superiority of OAGB; however, this difference was not statistically significant. This is likely caused by the OAGB-associated great hypoabsorptive characteristics, owing to the longer biliopancreatic limb, which bypasses a longer part of the jejunum, resulting in more efficient weight loss and alterations in the gastrointestinal immunological and hormonal profile [29]. It may alter intestinal flora and bile acids, with more accentuated distal bowel stimulation [30]. The current work showed that the favorable OAGB weight loss was also associated with relatively favorable rates of comorbidity remission. In line with our study, meta-analyses studies by Kaska et al. [31] and Magouliotis et al. [32] revealed that OAGB showed better weight loss and comorbidity remission rates than RYGB.

### Study limitations

This study has some limitations. The relatively small sample size and being a single-center study may limit the generalizability of the findings and the ability to detect more minor effects. Additionally, the cross-sectional retrospective design limits the ability to establish causal relationships between identified risk factors and the occurrence of marginal ulcers.

The a priori sample size calculation was based on previously published marginal ulcer incidences of 2.7% for RYGB and 5% for OAGB. However, the rates found in our study were considerably different at 3.2% and 19.4%, respectively. This difference likely reflects center-specific patient profiles, variations in surgical technique, and the rigor of postoperative endoscopic surveillance. Although the larger-than-expected effect size increased the post hoc statistical power of the study, the difference between the assumed and observed rates underscores a limitation in applying uniformly published incidence estimates across settings.

## Conclusion

Marginal ulcers occurred at a relatively high rate after bypass surgery in this cohort. OAGB is possibly associated with a lower incidence of MUs compared to RYGB. Although type 2 diabetes mellitus and the RYGB procedure were identified as significant predictors of MU after adjustment for confounding factors, these findings should be interpreted with caution, given the small sample size. Further studies with larger cohorts are needed to confirm these associations and provide a more comprehensive understanding of MU risk after bypass surgery.

## Supplementary Information

Supplementary Material 1.

## Data Availability

The datasets used and/or analyzed during the current study are available from the corresponding author.

## Abbreviations

BMI: Body Mass Index
DM: Diabetes Mellitus
EGD: Esophagogastroduodenoscopy
EWL%: Percentage of Excess Weight Loss
GI: Gastrointestinal
GIT: Gastrointestinal Tract
MU: Marginal Ulcer
NSAIDs: Non-Steroidal Anti-Inflammatory Drugs
OAGB: One-Anastomosis Gastric Bypass
PPI: Proton Pump Inhibitor
RYGB: Roux-en-Y Gastric Bypass
SPSS: Statistical Package for the Social Sciences
T2DM: Type 2 Diabetes Mellitus
TWL%: Percentage of Total Weight Loss
